# Wavelength and light-dose dependence in tumour phototherapy with haematoporphyrin derivative.

**DOI:** 10.1038/bjc.1985.146

**Published:** 1985-07

**Authors:** J. C. van Gemert, M. C. Berenbaum, G. H. Gijsbers

## Abstract

**Images:**


*Br. J. Cancer* (1985), **52**, 43–49

### Wavelength and light-dose dependence in tumour phototheraphy with haematoporphyrin derivative

J.C. van Gemert[1], M.C. Berenbaum[2] & G.H.M. Gijsbers[3]

[1]*Department of Medical Technology, St Joseph Hospital, Eindhoven, The Netherlands;* [2]*Department of Experimental Pathology, St Mary's Hospital Medical School, London W2, UK;* [3]*Laser Application and Information Centre Amsterdam (LAICA), Laboratory for Physical Chemistry Amsterdam, The Netherlands.*

**Summary** Red light (c. 630 nm) is almost universally used in tumour phototherapy as it is the most penetrating of the porphyrin excitation wavebands. However, measurements of tumour attenuation of light of different wavelengths and of the excitation spectrum of haematoporphyrin derivative *in vitro* suggested that green light might be more efficient than red in destroying thin tumours. Experimentally, we confirmed this for tumours up to ~1.2 mm thick, a depth exceeding that of most *carcinomas-in-situ.* The superiority of green light over red in terms of the illumination time required to produce equivalent depths of necrosis may extend to greater depths (3–4 mm) if the former is produced by an argon laser and the latter by an argon-pumped dye laser.

The relation between depth of necrosis $z_n$ and light dose $D$ is shown to be $z_n = \sigma_\lambda^{-1}\ln(D/\theta_\lambda)$ where $\sigma_\lambda$ is the attenuation coefficient for light at wavelength $\lambda$ and $\theta_\lambda$ the threshold light dose for producing necrosis at that wavelength. This logarithmic relationship suggests that it may be difficult to eradicate large tumours merely by increasing the light dose, and indicates the need for other approaches.

Tumour phototherapy depends on the ability of certain photosensitizing agents to localise in tumours, so that exposure of the tumour to sufficiently intense light of appropriate wavelength causes its rapid necrosis. The photosensitiser most widely used at present is "haematoporphyrin derivative" (HpD), a complex mixture of porphyrins (Bonnett *et al.*, 1980, 1981; Berenbaum *et al.*, 1982). HpD is excited at a number of discrete wavebands, most intensely by violet light at 400–410 nm (the Soret band), and with much less (and decreasing) intensity by bands at ~500–505 nm, 535–540 nm, 565–575 nm and 620–635 nm. Only the red waveband (620–635 nm) is used in tumour phototherapy as it has the greatest tissue penetration, an essential requirement in treating most human tumours, where light of adequate intensity may be needed through 1 cm or more of tissue.

However, the use of red light has disadvantages. First, generation of sufficiently intense red light requires (at present) use of a dye laser pumped by an ion laser, a costly and complicated arrangement. Second, the red waveband is the least efficient exciter of the porphyrin molecule.

Many human carcinomas do not demand use of highly penetrating light. For example, carcinoma-in-situ is usually well under 1 mm thick in most sites, and many early invasive carcinomas are only a few mm thick. The question therefore arises as to whether illumination at wavebands other than the red, with less penetration but more efficient excitation, might be more suitable for such tumours. Here, we compare the effectiveness of tumour phototherapy at 405 nm (in the Soret band), 514.5 nm (the main output of the argon laser) and 630 nm (the wavelength used in clinical phototherapy).

Correspondence: M.C. Berenbaum.
Reprint requests: M.J.C. van Gemert.
Received 22 October 1984; and in revised form 18 March 1984.

### Materials and methods

### *Haematoporphyrin derivative*

This was either (*a*) a 4 mg ml$^{-1}$ solution in 0.5% sodium hydrogen carbonate in PBS made from the solid, provided by Dr T.J. Dougherty, of the Roswell Park Memorial Institute or (*b*) the commercially available 5 mg ml$^{-1}$ solution ("Photofrin", Oncology Research and Development, Inc., Cheektowaga, New York).

### *Excitation spectra* in vitro

The fluorescence of HpD, 10 $\mu$g ml$^{-1}$ in 10% human serum in PBS was measured at 690 nm using the fluorimeter constructed by the Physical Chemistry group at the University of Amsterdam (Langelaar *et al.*, 1969) using a small path length to avoid distortion of the spectrum by HpD absorption.

© The Macmillan Press Ltd., 1985

*Light attenuation and scattering*

The tumour used for these measurements was the BA1112 rhabdomyosarcoma (Reinhold, 1966) obtained from Prof. H.S. Reinhold, Radiobiological Institute, TNO, the Netherlands. It was grown subcutaneously in WAG-Rij rats and used 6 weeks after implantation, when it was ~4 cm in diameter. Measurements of attenuation and scattering were made 2–3 h after excision, using diffuse light from a 10 cm integrating sphere, the interior of which was illuminated through a laterally placed aperture by light from a xenon arc used with a monochromator. Tumour slices 0.7–1.4 mm thick were held between 0.2 mm thick glass slides over a 1 cm aperture at the top of the sphere. Diffuse transmittance through them was measured by a photodiode (W34, AEG Telefunken) and diffuse reflectance by a second diode placed under an aperture in the bottom of the sphere. Correction for reflection from the glass surfaces was made using equations (4) and (5) of Kottler (1960).

Reflection and transmission were analysed in terms of absorption ($A$) and scattering ($S$) coefficients using Kubelka–Munk theory (cf Kottler (1960), equations (10)–(12)), as follows: Space irradiance $I_z$ at tumour depth $z$ is related to source intensity $I_0$ by

$$I_z = (1 - r_{sp})(1 + r_d) I_0 \cdot \exp(-\sigma z) \quad (1)$$

where $r_{sp}$ is the specular reflection coefficient of the tumour (typically 0.04–0.05), $r_d$ the diffuse reflectance from tumour thickness $d$ and $\sigma$ is the attenuation coefficient given by $\sigma = \sqrt{A(A+2S)}$ (Ishimaru, 1978). With our data (Table I) and assuming a "worst case" tumour depth of 8 mm, $r_d$ is ~0.09 at 630 nm, 0.12 at 514.5 nm and <0.08 at 405 nm. Thus, $(1 - r_{sp})(1 + r_d) \sim 1$ at all wavelengths used, and we accordingly used the approximation

$$I_z \simeq I_0 \cdot \exp(-\sigma z). \quad (2)$$

*Tumour necrosis*

The PC6 plasma cell tumour, obtained originally from the Chester Beatty Research Institute, was passaged s.c. in BALB/c female mice, and used at diameters up to 10–12 mm and depths up to 6 mm, 10–16 days after injection of $10^6$ cells. HpD was given in a fixed i.v. dose of 0.4 mg 10 g$^{-1}$ body weight. Twenty-four hours later, the skin overlying the tumour was shaved and depilated, mice were anaesthetised and the tumours illuminated at wavebands 405 ± 18.6, 514.5 ± 18.6 or 630 ± 18.6 nm using a 900 W xenon arc fitted with a f/3.4 grating monochromator (Applied Photophysics Limited), with a slit width of 8 mm and a spectral band-pass of 4.65 nm mm$^{-1}$. Light intensity at the tumour surface, measured with a thermopile (Model 14BT, with indicator 154BT, Laser Instrumentation Limited), varied in different experiments over the ranges 90–107 mW cm$^{-2}$ at 405 nm, 100–108 mW cm$^{-2}$ at 514.5 nm and 69–76 mW cm$^{-2}$ at 630 nm, and light dose was varied by adjusting the time of exposure. The depth of tumour necrosis was measured 24 h after illumination as described previously (Berenbaum *et al.*, 1982).

### Results

*Photophysical parameters*

Table I shows relative excitation efficiencies for HpD in 10% serum of light at 630, 514.5 and 405 nm, and the values of $A$, $S$ and $\sigma$ for tumour (rhabdomyosarcoma). $A$ and $S$ at 630 and 514.5 nm were measured as described above. The value for $A$ at 405 nm was calculated as follows. At 405 and 514.5 nm, tissue absorption is dominated by its blood content, but this has a negligible effect at 630 nm (for oxygenated blood, $A = 4$ cm$^{-1}$ at 630 nm, 105 cm$^{-1}$ at 514.5 nm and 2600 cm$^{-1}$ at 405 nm (Ishimaru, 1978; van Gemert & Hulsbergen Henning, 1981)). Thus, the fractional blood volume of the tumour may be estimated from the values for $A$ at 514.5 and 530 nm (Table I) as (4.5–2.2)/105 = 0.022, and its absorption coefficient at 405 nm as 0.022 × 2600 = 57.2 cm$^{-1}$. The value of $S$ at 405 nm was calculated using published data for wavelength dependence of scattering (Anderson & Parrish,

**Table I** Relative excitation efficiencies ($E$) and absorption ($A$), scattering ($S$) and attenuation ($\sigma$) coefficients

| *Waveband (nm)* | $E^a$ | $A^b$ ($cm^{-1}$) | $S^b$ ($cm^{-1}$ | $\sigma^c$ ($cm^{-1}$) |
|---|---|---|---|---|
| 630 ± 18.6 | 1 | 2.2 | 5.0 | 5.2 |
| 514.5 ± 18.6 | 2.37 | 4.5 | 7.4 | 9.3 |
| 405 ± 18.6 | 19.7 | 57.2[d] | 11.0[e] | 67.3 |

[a]Measured on HpD, 10 µg ml$^{-1}$ in 10% human serum in PBS. Arbitrary units, based on $E = 1$ for 630 ± 18.6 nm, taking into account a gaussian form for the wavebands, with means at 630, 514.5 and 405 nm and 50% values at ± 18.6 nm on either side of the mean.

[b]Measured on the BA1112 tumour.

[c]$\sigma = \sqrt{A(A+2S)}$ (Ishimaru (1978), equations (10-6) and (10-14).

[d]Calculation based on an estimated 2.2% (v/v) blood content of tumour and data for blood from Ishimaru (1978) and van Gemert & Hulsbergen Henning (1981) (see text).

[e]Extrapolated value using the wavelength dependence of dermal scattering (Anderson & Parrish, 1981) for scaling (see text).

1981), who found that values for $S$ at 630, 514.5 and 405 nm were in the ratios 6:9:14. These ratios, with our experimentally determined values of 5 and 7.4 $cm^{-1}$ at the two longer wavelengths indicate a value for $S$ of c. 11 $cm^{-1}$ at 405 nm.

The resulting attenuation coefficient at 405 nm ($\sigma = 67.3\ cm^{-1}$, Table I) agrees with data obtained by Eichler *et al.* (1977) for other tissues, which are in the range 55–70 $cm^{-1}$. Our value of 5.2 $cm^{-1}$ for $\sigma$ for tumour at 630 nm is close to the value of 6.3 $cm^{-1}$ found for a human lung carcinoma by Svaasand *et al.* (1981). Further, measurements (not presented here) on a rat urothelial tumour used previously (Gijsbers *et al.*, 1984) yielded results very similar to those of Table I. The data in Table I may therefore represent typical values for a range of normal and neoplastic tissues.

*Tumour necrosis*

Figure 1 shows that there is a more or less linear relation between depth of necrosis and the logarithm of the light dose. Red light has the steepest dose-effect curve (slope 0.20 when dose is measured in natural logarithms) and the highest threshold for damage ($\sim$4.2 J $cm^{-2}$), violet light has the shallowest curve (slope $\sim$0.023) and the lowest threshold (about 0.8 J $cm^{-2}$) and green light is intermediate in both respects (slope 0.11 and threshold 2.5 J $cm^{-2}$). Whereas red light is the most efficient of the three wavebands in causing tumour necrosis at depths exceeding $\sim 1.2 \pm 0.5$ mm, green light is more efficient at lesser depths. Violet light is the most efficient only at depths less than $\sim 0.2 \pm 0.1$ mm.

### Discussion

The relation between light dose and the extent of tissue damage is of great importance in tumour phototherapy. When tissues are illuminated *in vivo*, light intensity falls more or less exponentially with tissue depth (Eichler *et al.*, 1977; Svaasand *et al.*, 1981). However, the rapid necrosis that ensues in photosensitised tissues, due to formation of chemically reactive singlet oxygen, does not. Instead, there is a zone of completely necrotic tissue and an abrupt transition to deeper tissue that appears histologically undamaged (Berenbaum *et al.*, 1982, and Figure 2). This suggests that, for

**Figure 1** Relation between light dose and depth of necrosis in the PC6 tumour at 630 ± 18.6 nm (□), 514.5 ± 18.6 nm (○) and 405 ± 18.6 nm (●). The number of tumours used is indicated by each mean. Vertical bars represent s.d. Linear regressions using the individual measurements are represented by the dashed curves: (---) 405 ± 18.6 nm; (-·-·) 514 ± 18.6 nm; (-··-··) 630 ± 18.6 nm.

**Figure 2** PC6 tumour 48 h after injection of HpD 40 mg kg$^{-1}$ and 24 h after exposure of tumour to 10 J cm$^{-2}$ light at 630 ± 18.6 nm. The superficial aspect of the tumour is uppermost. Note the abrupt transition from necrotic tumour above to apparently undamaged tumour below. (H&E, × 125).

production of necrosis, there is a threshold local dose of singlet oxygen (the threshold varying with the tissue), and that cells either die or survive, according to whether the local dose exceeds or is less than this. In this context, the "dose" of singlet oxygen may be defined as the product of its local concentration ($^1O_2$) and the time $t$ over which that concentration is maintained.

No information is available about singlet oxygen levels or excitation behaviour in tissues during phototherapy, so some reasonable assumptions are necessary in analysing the results. First, as the lifetime of singlet oxygen is negligible compared with the time of exposure to light in phototherapy, we may assume that the duration of exposure of tissues to singlet oxygen is the same as that of their exposure to light. Second, the relative excitation efficiencies for HpD in tissues are assumed to be the same as that in 10% serum. Third, as incident light activates the porphyrin molecule but does not influence the subsequent distribution of energy between the paths leading, on the one hand, to fluorescence and, on the other, to singlet oxygen production (Dougherty *et al.*, 1976), we assume that the ratio of distribution of energy between these two paths is fixed, and thus that relative fluorescence excitation efficiencies at different wavelengths and relative efficiencies for generating singlet oxygen should be proportional to each other. We therefore assume that the local dose ($^1O_2$) $t$ of singlet oxygen is proportional to relative fluorescence excitation efficiency $E_\lambda$ at wavelength $\lambda$ (Table I) and to total light dose $D$ (which is the product of local space irradiance $I$ and time $t$ of illumination), i.e.,

$$(^1O_2)t = kE_\lambda D \qquad (3)$$

Here $k$ is a constant that depends on the level of HpD in the tissue but, as HpD dose and the interval between its administration and exposure to light were fixed in these experiments, we may assume a fixed HpD concentration in tumours at the time of illumination.

From equation (3), threshold light doses $\theta_{\lambda 1}$, $\theta_{\lambda 2}$ needed to generate the threshold dose of singlet oxygen at wavelengths $\lambda_1$ and $\lambda_2$ are related by:

$$kE_{\lambda 1}\theta_{\lambda 1} = kE_{\lambda 2}\theta_{\lambda 2} \quad (4)$$

and therefore

$$\theta_{\lambda 1} = \frac{E_{\lambda 2}}{E_{\lambda 1}}\theta_{\lambda 2}$$

That is, for light at different wavelengths, threshold doses for producing tissue necrosis are inversely proportional to their relative excitation efficiencies for fluorescence. (We do not complicate matters here by considering possible repair mechanisms that would have dose-related effects and so necessitate consideration of $I$ and $t$ separately, as we have not found depth of necrosis produced by our light source *in vivo* to be affected appreciably by variations in light intensity and duration that keep total dose fixed (Berenbaum, unpublished). The depth of necrosis $z_n$ is that at which the threshold dose of singlet oxygen is produced, i.e., the depth at which attenuation reduces the light dose $D_0$ at the surface (where $D_0 = I_0 t$) to the threshold dose. This may be calculated from equation (2).

$$I_{z_n}t = I_0 t \exp(-\sigma z_n) = \theta$$

Therefore,

$$z_n = \frac{1}{\sigma}\ln\left[\frac{I_0 t}{\theta}\right]. \quad (5)$$

That is, depth of necrosis should be proportional to the logarithm of light dose, a prediction borne out by Figure 1. Equation (5) shows that a graph of depth of necrosis as a function of $\log_e$ light dose should be a straight line with slope of $\sigma^{-1}$ and intercept $\theta$ on the dose axis.

A comparison between the experimental dose-effect curves of Figure 1 and those calculated from Table I using equations (4) and (5) is shown in Figure 3. Calculations were based on the experimentally measured threshold at 630 nm, as this would be least affected by variations in tumour content of blood, whether oxygenated or not. The photophysical parameters ($A$ and $S$) and depth of necrosis were perforce measured in two different tumours, but both are solid, homogeneous, and non-pigmented, and there was no *a priori* reason to expect their photophysical properties to differ

**Figure 3** Predicted dose-response curves (solid lines) for light at 630 ± 18.6 nm, 514.5 ± 18.6 nm and 405 ± 18.6 nm. Calculations according to equations (4) and (5), using the relative excitation efficiencies and attenuation coefficients of **Table 1** and the observed threshold of 4.2 J cm$^{-2}$ at 630 nm. The experimentally determined curves of **Figure 2** are inserted for comparison (dashed curves as in **Figure 2**).

markedly. In fact, as mentioned above, a variety of normal and neoplastic tissues are found to have rather similar attenuation coefficients. Considering the uncertainties in measuring the optical properties of tumours and the large variations inherent in producing and measuring tumour necrosis, we think that the level of agreement found between calculated and observed measurements (Figure 3) suggests that our overall approach is correct, although much refinement is needed, both theoretically and experimentally. In particular, Kubelka–Munk theory has limited validity for the collimated light we used to treat tumours, and other analytic methods (Svaasand *et al.*, 1981) may be more appropriate.

*Clinical implications*

Figure 1 shows that the dose-effect curves for tumour necrosis produced by red and green light respectively intersect at a tissue depth of ~1.25 mm, green light being more efficient at lesser depths. However, these dose-effect curves do not fully reveal the potential advantage of green light for thin tumours with currently available light sources. Light at 630 nm is at present generally produced by a dye laser pumped by an argon laser, but the conversion efficiency is at best 20–25%. More than half the argon output is in the green (514.5 nm), the rest being mainly blue (488 nm). Therefore, 2–3 times as much power in the green may be obtained using the argon laser directly as may be obtained in the red from the dye laser. Thus, in terms of the illumination time needed to produce equivalent depths of necrosis, a more realistic comparison of the two wavebands would be made by transposing the dose-effect for green light to the left by a shift corresponding to a 2-to-3-fold reduction in dose. The curve for red light and the transposed curve for green would intersect at a depth of ~3.3 mm. An additional effect would be produced by the blue argon output (488 nm), which has about the same (HpD) excitation efficiency as light at 514.5 nm and nearly the same tissue penetrance. Thus our findings suggest that, for tumours of up to 3.3 mm depth, and probably more, shorter illumination times would be entailed by using an argon laser directly rather than a red-emitting dye laser pumped by it. This depth encompasses that of tumours more extensive than carcinoma-in-situ, for example, many microinvasive carcinomas of the bladder (Farrow & Utz, 1982).

The argon laser is thus likely to be most useful where the tumour is thin and a large area requires illumination, for instance, in widespread carcinoma-in-situ of the bladder. Ideally, the whole bladder surface should be illuminated as local treatment is very likely to be followed by recurrence in the untreated area (Tsuchiya *et al.*, 1983; Benson *et al.*, 1983), and delivery of an adequate dose of red light to the whole mucosa of the distended bladder, 2–300 $cm^2$, with dye lasers now available may be prohibitively time-consuming. Use of argon laser with at least 2–3 times as much output at wavelengths that are more efficient than red in destroying superficial tumours may be preferable. Bellnier *et al.*, (1984, 1985) have independently drawn the same conclusion, after finding that green and red laser light at equal doses and dose-rates were equally effective in destroying thin ($\leq$2.5 mm deep) tumours.

The question arises as to how far these findings may be extrapolated to man. Our mice (17–22 g) received 40 $mg\,kg^{-1}$ HpD, equivalent on a surface area basis to about 3 $mg\,kg^{-1}$ in man, a dose within the clinical range (2.5–5.0 $mg\,kg^{-1}$). Such measurements as have been made suggest that 100–360 $J\,cm^{-2}$ of light at 630 nm produces 5–10 mm depth of necrosis in human tumours (Dougherty *et al.*, 1985). Extrapolation of the curve for 630 nm in Figure 1 shows that, in our model, these doses would produce 6.5–9 mm necrosis. Thus, agreement is surprisingly good.

The log-linear relation between light dose and depth of necrosis also has important implications. If the curve for red light in Figure 2 may be extrapolated, it suggests that it would require about 500 $J\,cm^{-2}$ at 630 nm to cause necrosis to a depth of 1 cm, which is feasible with current light sources. However, producing necrosis to a depth of ~1.5 cm would require ~6000 $J\,cm^{-2}$, necessitating either impracticably long illumination times (a few hours) or light intensities at which thermal effects would predominate and selectivity due to photo-sensitisation would virtually disappear. Therefore progress in treating large tumours cannot depend solely on developing light sources of greater power but also on (*a*) developing methods for distributing light more uniformly throughout the tumour and (*b*) finding photosensitisers that are excited efficiently at longer and more penetrating wavelengths, thus in effect increasing the slope of the tumour necrosis dose-effect curve.

M.C. Berenbaum is supported by the Medical Research Council. We are grateful to Dr T.J. Dougherty for a gift of HpD and to Prof. H.S. Reinhold for providing the rhabdomyosarcoma. We gratefully acknowledge assistance by Mrs A. Goldsmith in London, and by Ruud Verdaasdonk and Gert Schets in the Netherlands. We are indebted to Dr Jan Langelaar for discussions in the early stages of the work and for hospitality in the Physical Chemistry Department (Amsterdam) while the optical measurements were made.

**References**

ANDERSON, R.R. & PARRISH, J.A. (1981). The optics of human skin. *J. Invest. Dermatol.*, **77**, 13.

BELLNIER, D.A., LIN, C.-W. & PROUT, G.R. Jr. (1984). Treatment of bladder carcinoma with 514 nm light plus HpD: an approach to whole bladder photoradiation therapy. *J. Urol.*, **131**, 110A.

BELLNIER, D.A., PROUT, G.R. Jr. & LIN, C.-W. (1985). Effect of 514.5 nm argon ion laser irradiation on hematoporphyrin derivative-treated bladder tumor cells *in vitro* and *in vivo*. *J. Natl Cancer Inst.* (in press).

BENSON, R.C., Jr., KINSEY, J.H., CORTESE, D.A., FARROW, G.M. & UTZ, D.C. (1983). Treatment of transitional cell carcinoma of the bladder with hematoporphyrin derivative phototherapy *J. Urol.*, **130**, 1090.

BERENBAUM, M.C., BONNETT, R. & SCOURIDES, P.A. (1982). *In vivo* biological activity of the components of haematoporphyrin derivative. *Br. J. Cancer*, **42**, 571.

BONNETT, R., RIDGE, R.J., SCOURIDES, P.A. & BERENBAUM, M.C. (1980). Haematoporphyrin derivative, *J. Chem. Soc. Chem. Comm.*, 1199.

BONNETT, R., RIDGE, R.J., SCOURIDES, P.A. & BERENBAUM, M.C. (1981). On the nature of "haematoporphyrin derivative". *J. Chem. Soc., Perkin* **I**, 3135.

DOUGHERTY, T.J., GOMER, C.J. & WEISHAUPT, K.R. (1976). Energetics and efficiency of photoinactivation of murine tumor cells containing hematoporphyrin. *Cancer Res.*, **36**, 2330.

DOUGHERTY, T.J., WEISHAUPT, K.R. & BOYLE, D.G. (1985). Photodynamic therapy and cancer. In: *Principles and Practice of Oncology*, (Eds. de Vita *et al.*), 2nd ed., Philadelphia: Lippincot.

EICHLER, J., KNOPF, J. & LENZ, H. (1977). Measurements on the depth of penetration of light (0.35–1.0 $\mu$m) in tissue. *Rad. Environm. Biophys.*, **14**, 239.

FARROW, G.M. & UTZ, D.C. (1982). Observations on microinvasive transitional cell carcinoma of the urinary bladder. In: *Minimal Invasive Cancer* (*Microcarcinoma*), Clin. Oncol., **1**, 608.

GEMERT, M.J.C. VAN & HULSBERGEN HENNING, J.P. (1981). A model approach to laser coagulation of dermal vascular lesions. *Arch. Dermatol. Res.*, **270**, 429.

GIJSBERS, G.H.M., GEMERT, M.J.C. VAN, BREEDERVELD, D., LANGELAAR, J. & BOON, T.A. (1984). In vivo fluorescence excitation spectra of hematoporphyrin derivative (HpD). In: *Porphyrins in Tumor Phototherapy*. (Eds. Andreoni & Cubeddu), Plenum Publishing Corporation: New York, p. 339.

ISHIMARU, A. (1978). *Wave Propagation and Scattering in Random Media*, Vol. 1. Single scattering and transport theory. Academic Press: New York.

KOTTLER, F. (1960). Turbid media with plane-parallel surfaces. *J. Opt. Soc. Am.*, **50**, 483.

LANGELAAR, J., DEVRIES, G.A. & BEBELAAR, D. (1969). Sensitivity improvements in spectrofluorimetry. *J. Sci. Instrum.*, **46**, 149.

REINHOLD, H.S. (1966). Quantitative evaluation of the radiosensitivity of cells of a transplantable rhabdomyosarcoma in the rat. *Eur. J. Cancer*, **2**, 33.

SVAASAND, L.O., DOIRON, D.R. & PROFIO, A.E. (1981). Light distribution in tissue during photoradiation therapy. *Tech. Report, University of Southern California, Institute for Physics and Imaging Science*.

TSUCHIYA, A., OBARA, N., MIWA, M., OHI, T., KATO, H. & HAYATA, Y. (1983). Hematoporphyrin derivative and laser photoradiation in the diagnosis and treatment of bladder cancer. *J. Urol.*, **130**, 79.


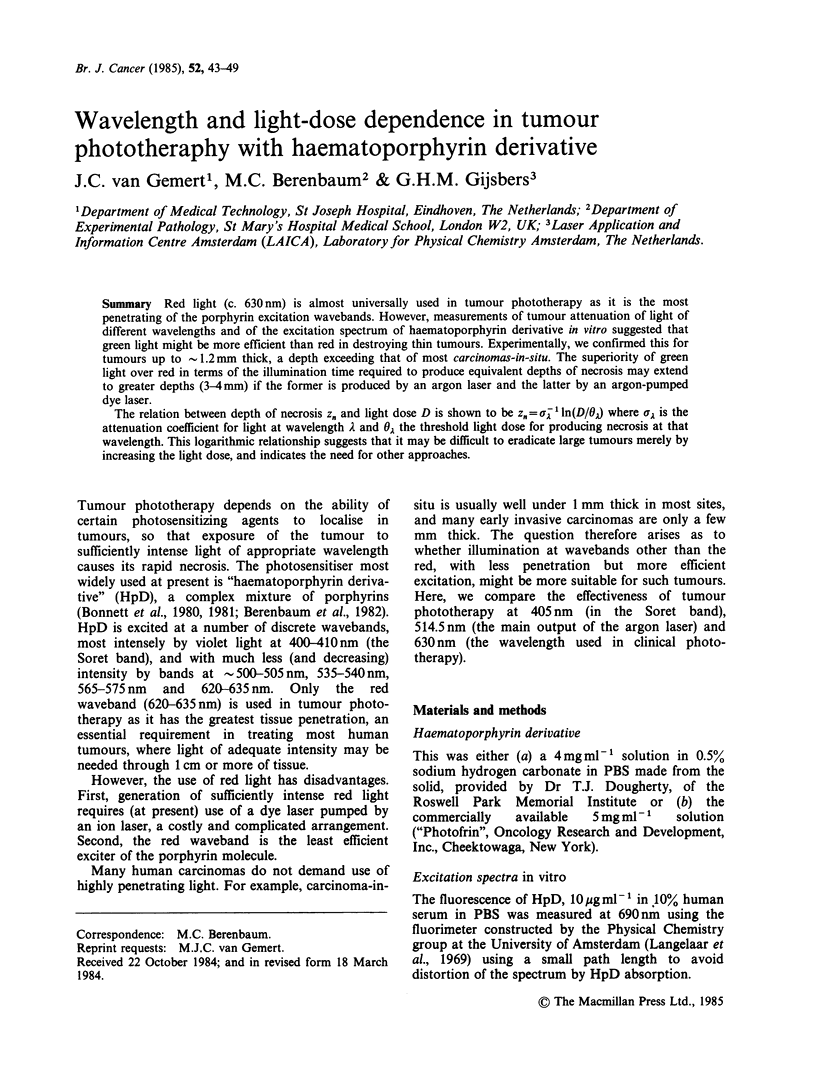

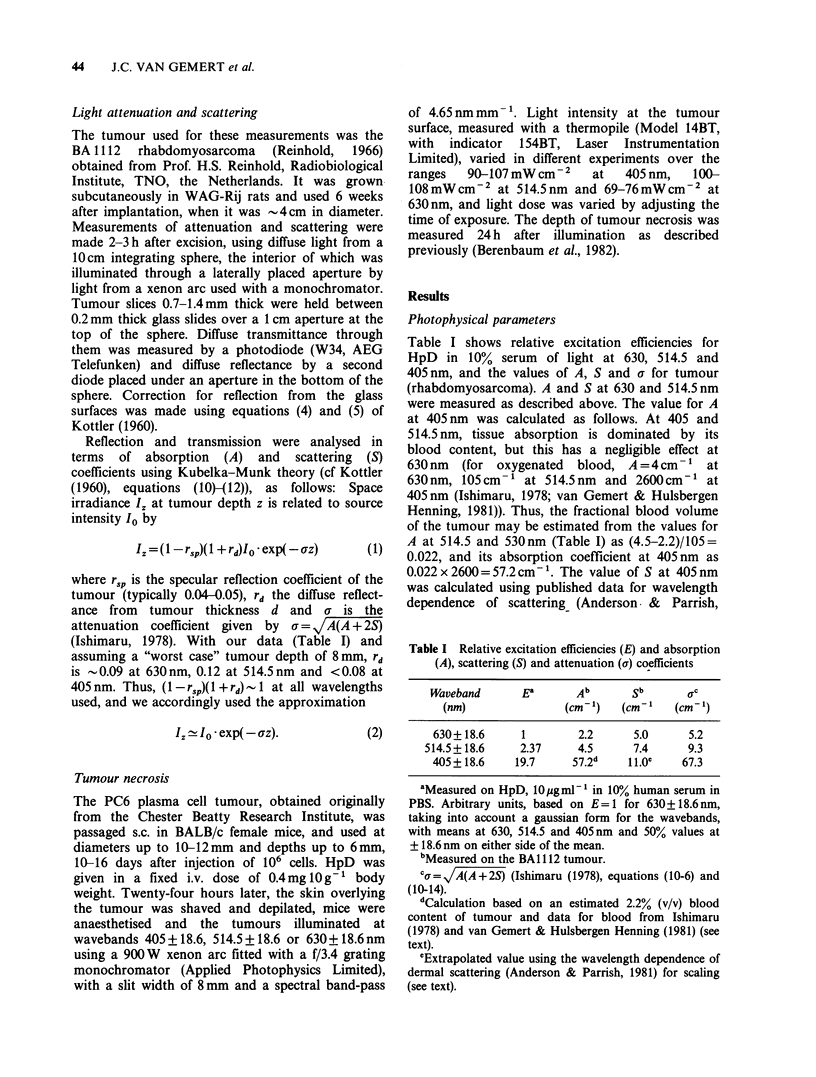

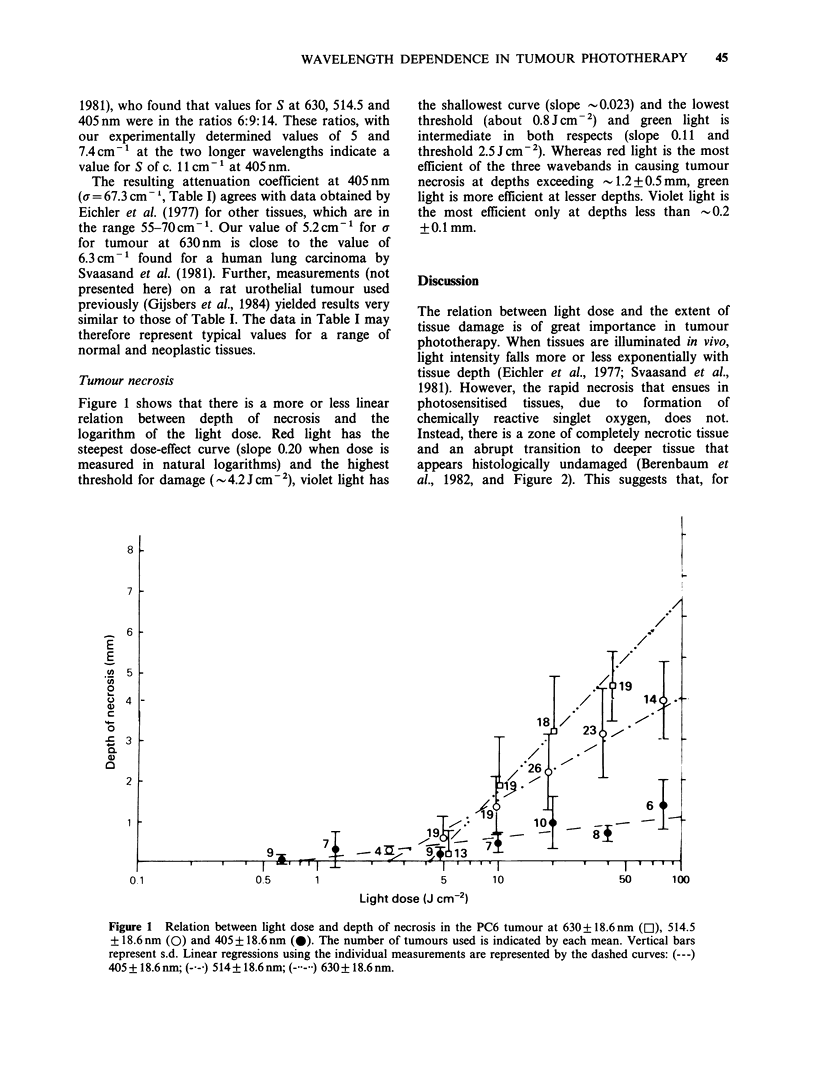

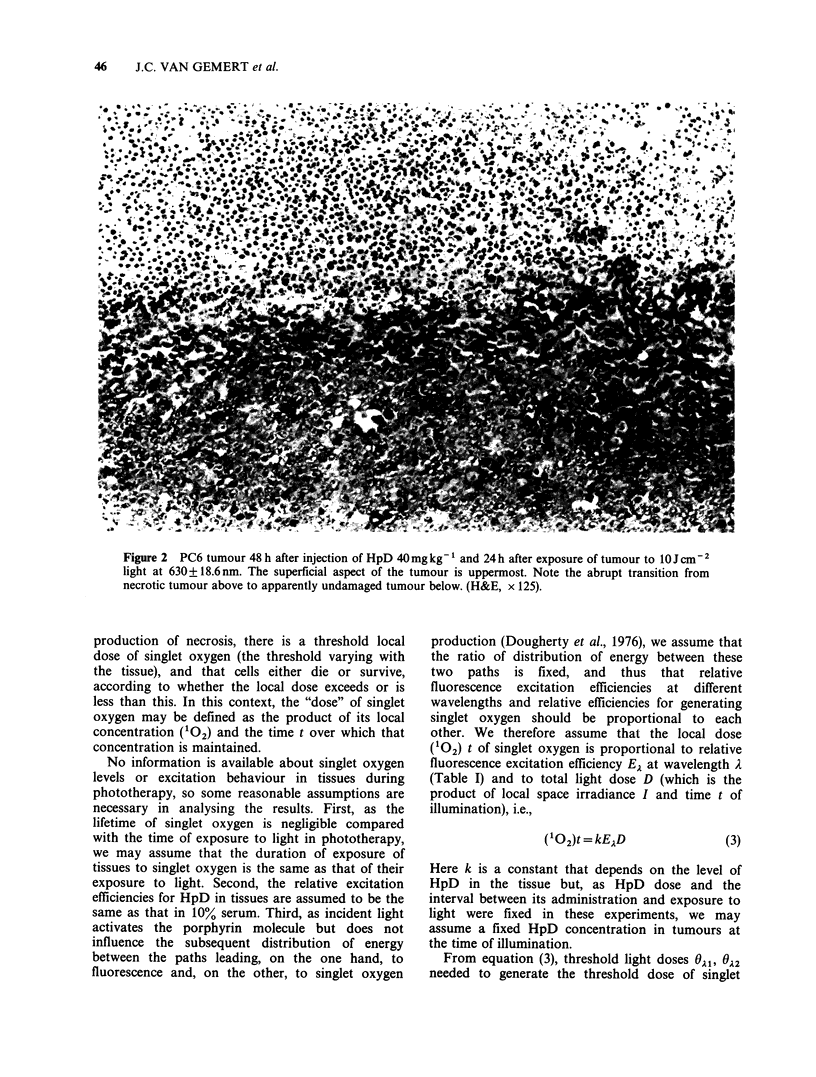

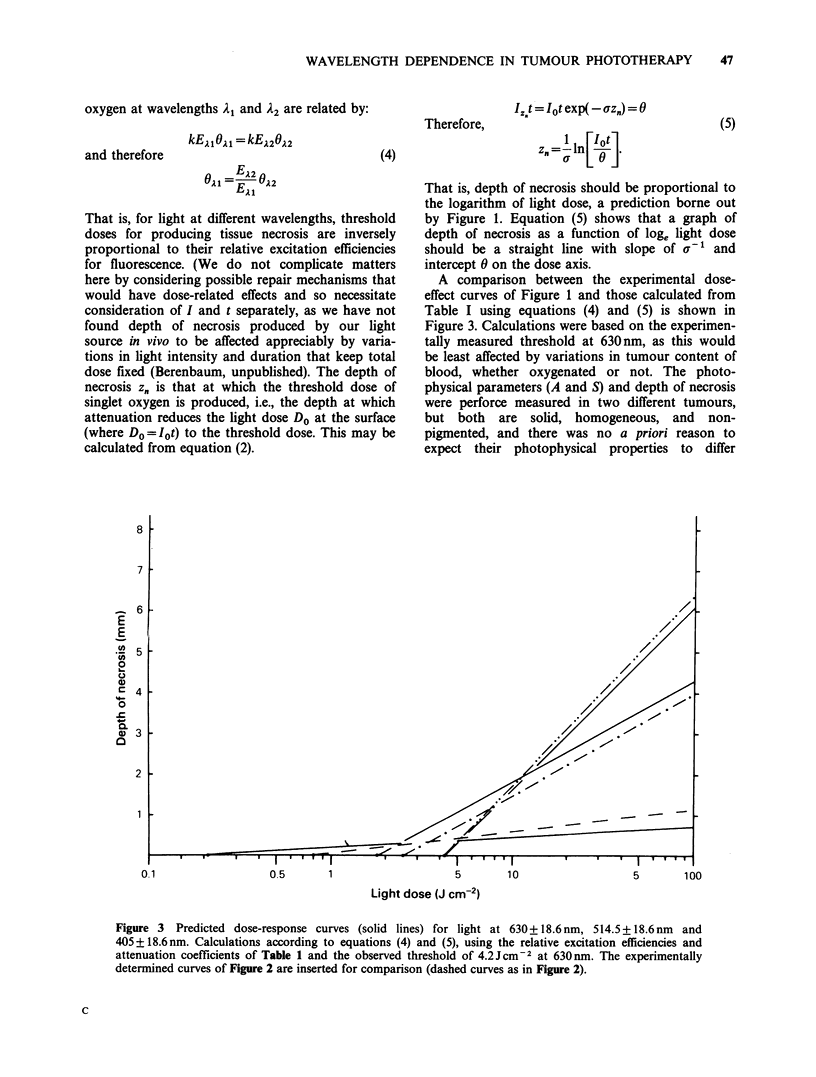

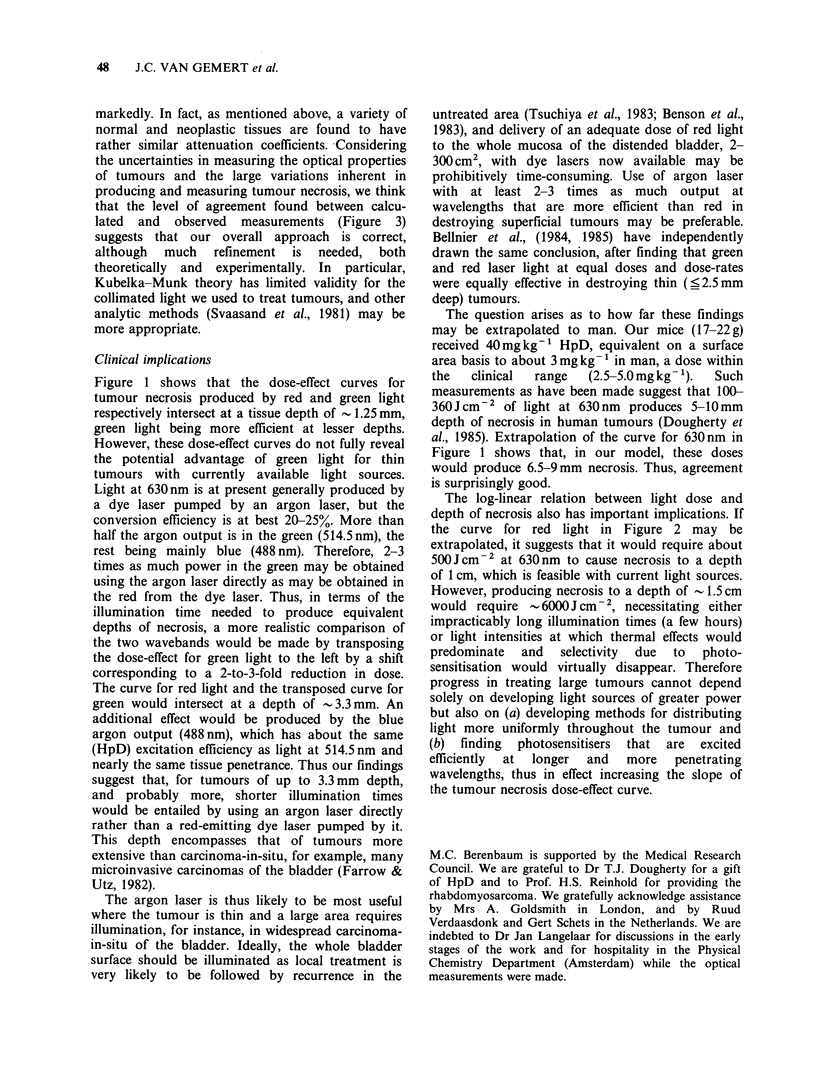

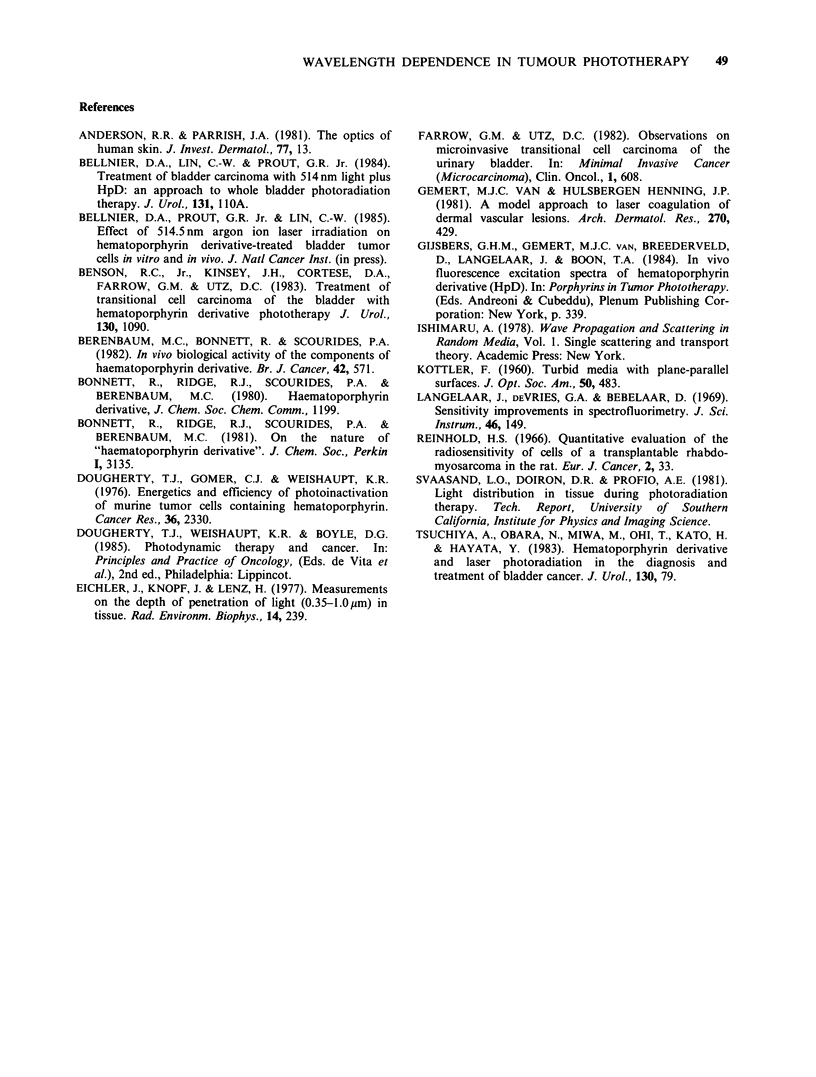


## References

[OCR_00608] Anderson R. R., Parrish J. A. (1981). The optics of human skin.. J Invest Dermatol.

[OCR_00623] Benson R. C., Kinsey J. H., Cortese D. A., Farrow G. M., Utz D. C. (1983). Treatment of transitional cell carcinoma of the bladder with hematoporphyrin derivative phototherapy.. J Urol.

[OCR_00630] Berenbaum M. C., Bonnett R., Scourides P. A. (1982). In vivo biological activity of the components of haematoporphyrin derivative.. Br J Cancer.

[OCR_00646] Dougherty T. J., Gomer C. J., Weishaupt K. R. (1976). Energetics and efficiency of photoinactivation of murine tumor cells containing hematoporphyrin.. Cancer Res.

[OCR_00658] Eichler J., Knof J., Lenz H. (1977). Measurements on the depth of penetration of light (0.35--1.0 microgram) in tissue.. Radiat Environ Biophys.

[OCR_00692] Langelaar J., de Vries G. A., Bebelaar D. (1969). Sensitivity improvements in spectrophospho-fluorimetry.. J Sci Instrum.

[OCR_00697] Reinhold H. S. (1966). Quantitative evaluation of the radiosensitivity of cells of a transplantable rhabdomyosarcoma in the rat.. Eur J Cancer.

[OCR_00708] Tsuchiya A., Obara N., Miwa M., Ohi T., Kato H., Hayata Y. (1983). Hematoporphyrin derivative and laser photoradiation in the diagnosis and treatment of bladder cancer.. J Urol.

[OCR_00669] van Gemert M. J., Henning J. P. (1981). A model approach to laser coagulation of dermal vascular lesions.. Arch Dermatol Res.

